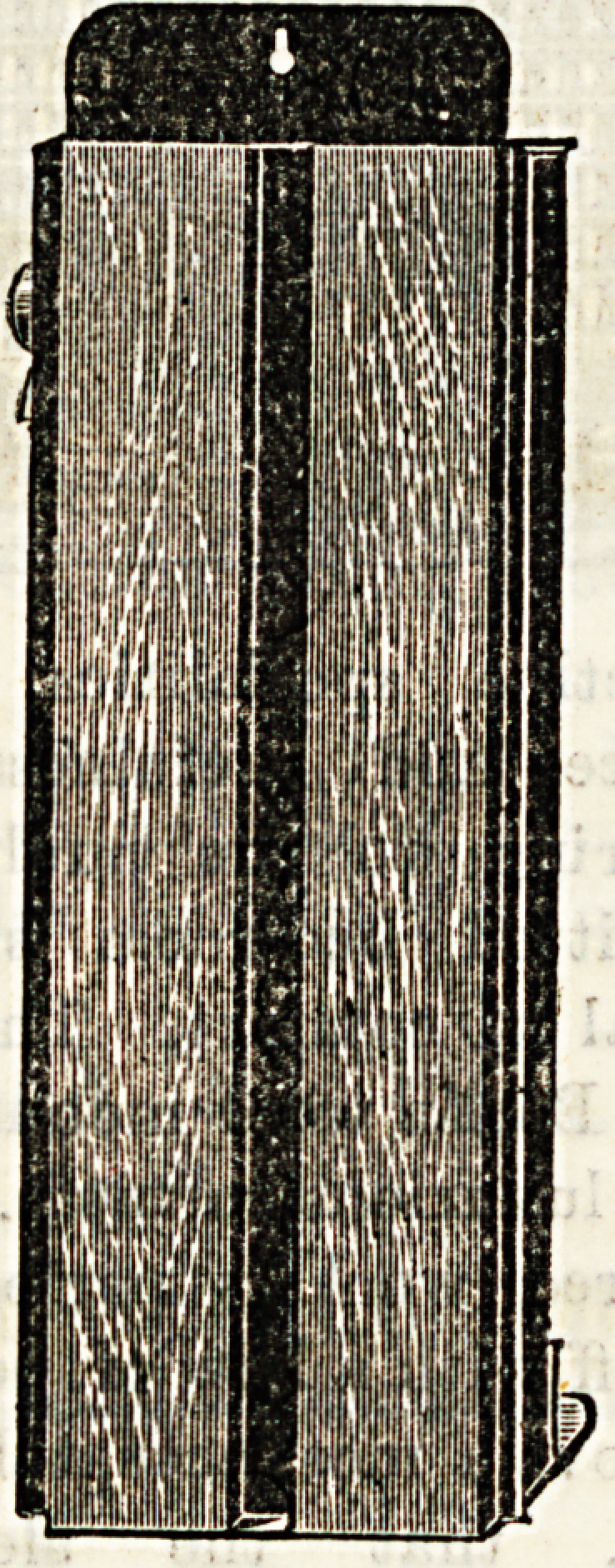# The Hospital Nursing Supplement

**Published:** 1894-12-22

**Authors:** 


### "The Hospital" Nursing Mirror.

Being the Extra Nursing Supplement of "The Hospital" Newspaper.

[Contributions for this Supplement should be addressed to the Editor, The Hospital, 428, Strand, London, W.C., and should have the word "Nursing" plainly written in left-hand top corner of the envelope.]

### News from the Nursing World.

### OUR CHRISTMAS GREETING.

A Happy Christmas to you, one and all, dear nurses and fellow-workers. It really does not seem long since The Hospital last greeted you in the same strain. Again we have to thank you for kind interest, encouragement, support, and sympathy. We have catered for your instruction and amusement, and your appreciation of the "Supplement" is welcomed by us. We hope that nurses at home and abroad will continue to send us such items of news regarding their work and surroundings as may be helpful to others. Busy workers are apt to think little things are "not worth writing about;" forgetting that details which soon become familiar to themselves are new and strange to those at home. Much mutual help can be given by a little thought of what will please and help sister nurses.

### OUR PRINCESS.

The nurses belonging to the Royal National Pension Fund will hear with pleasure that Her Royal Highness the Princess of Wales hopes, in the course of next year, to herself present them with the badge and armlet specially designed for them. "Our Princess" has long wished to identify with herself the nurses belonging to the Fund, of which she is the Royal President, and the badge selected is in the Danish colours. The groundwork is of rich crimson twilled silk ribbon, on which is a narrow white edge, the badge or shield bears a monogram in white embroidery, surmounted by a crown. Lady Rothschild, President of the Benevolent Branch of the R.N.P.F., has taken a most kindly interest in the matter, and the armlets and badges are made up by children in the Jewish schools.

### METROPOLITAN AND PROVINCIAL BAZAARS.

What an epidemic of bazaars there has been this month, and what variations there have been in the degrees of financial success attained by them. The one which ought to have realised big profits was surely the much-talked-of enterprise at the Grafton Galleries, where Princess Christian with unprecedented devotion acted the part of a cheery and gracious saleswoman for three consecutive days at her presidential stall. It is, therefore, somewhat surprising to learn that even H.R.H.'s energy and cordial co-operation failed to make the bazaar anything like so profitable as was desired. Some £900 with, say, £200 or £300 deducted for expenses, is a small part of the sum hoped for by the association already so deeply indebted to its Royal patron for liberal support. Provincial bazaars for charities frequently produce such large sums that it appears as if this kind of thing better suited provincial than metropolitan tastes. At Southport, for instance, the Grand Bazaar, held last month in the Municipal Buildings, brought in £5,381.

### THE LONDON HOSPITAL.

With such elaborate "Higher Education" schemes for the nurses of the future discussed on all sides, it is pleasant to find the nurses of to-day are still being well grounded in practical work. At the London Hospital, for instance, we learn that the results of the bandaging examinations have proved particularly encouraging, whilst the classes in sickroom cookery have had admirable results. The consent of the governors to largely increase the accommodation for their nurses is a most satisfactory proof of their interest in their staff. The progress made at the London Hospital Training School can hardly be estimated by those who cannot remember the quarters and the food which were thought "quite good enough for nurses" before the *régime* of the present indefatigable matron. We should advise our readers to go to the "Children's Trees" at 2.30 on December 31st, and to inspect for themselves the management of the largest general hospital in London. Invitations to the fête can be obtained by application to the Matron, London Hospital, Whitechapel.

### A GOOD EXAMPLE.

The deep interest felt in the future as well as the present well-being of the nursing staff is evinced in many ways at Guy's Hospital. A fresh proof of kindly forethought is shown in the following extract from the minutes of a Court of Committees held on December 12th, 1894: "Resolved, that it be intimated to the Matron for the information of the nurses that all nurses who, after the completion of their three years' training, may continue in the service of the hospital, must subscribe to the National Pension Fund, according to the scheme approved by the Treasurer in April, 1893, and that all present sisters and staff nurses are advised to adopt the same course, it being the intention of the Governors to refuse applications for pensions and allowances outside those so liberally provided for in the above-named scheme."

### QUESTIONS OF INTEREST.

The Local Government Board has been appealed to by the matron of the Lambeth Infirmary to abolish the office of superintendent of nurses in that institution. Pending the inquiry to be held at Lewisham Infirmary the matron, whom the Guardians have "suspended," has temporarily left the institution. The decision of the Local Government Board in both instances is awaited with anxiety by all interested in the subject of trained nursing for workhouse infirmaries.

### IN SOUTH LONDON.

St. Olave's District Nursing Association provides nurses for the sick poor in their own homes in Bermondsey, Rotherhithe, St. Olave, St. John, and St. Thomas's parishes. Affiliated with the Queen's Jubilee Institute, the staff consists of a superintendent and four fully-trained nurses. The unsectarian nature of the work of the institute was commended by the speakers at the recent annual meeting, one of whom spoke of the work done, as "living religion."

### NURSING IN MANCHESTER.

The Sick Poor and Private Nursing Institution held its twenty-ninth annual meeting at Manchester the other day, under the presidency of the Lord Mayor. Thirty-five fully-trained nurses devote themselves to the district work, and a house outside the town is set apart as a quarantine home for those who have been engaged with infectious cases. During the year 6,092 patients had been attended, to whom 137,000 visits had been paid.

### DISTRICT NURSING FOR DAVENTRY.

At Daventry a public meeting has been held to consider the establishment of a nursing club for the town and villages around. Miss Hughes, superintendent of the Central Training Home for Queen's Nurses, Bloomsbury, was present on the occasion, and gave a lucid description of the requirements of such associations. Finally a representative committee was chosen to go into the matter.

### QUEEN'S NURSES IN DUBLIN.

The St. Lawrence's Catholic Home for Nurses held its annual meeting in Dublin on the 6th inst. There was a very large attendance at the meeting, which was presided over by the Lord Mayor, and the record of the work accomplished during the year was excellent. Miss Dunne, head of the Irish Branch of the Queen's Jubilee Institute, was amongst the speakers, and her remarks on the advantage of trained nursing for the sick poor received the attention they merited.

### IMPROVED AND ENLARGED.

The annual meeting of the supporters of the Dublin Orthopædic Hospital was held on the 12th inst., the Lord Chancellor of Ireland presiding. The hospital has been enlarged during the past year by the acquisition of an adjoining house, and a well-equipped gymnasium has also been added. Patients and nurses alike have benefited by a long sojourn in the country, the thoughtful kindness of the Countess of Mayo having placed Johnstown House, co. Kildare, at the disposal of the hospital authorities, together with a supply of milk and vegetables.

### YOUNG GIRLS.

Serious complaints against the work and hours of the nurses at the Newcastle Royal Infirmary are appearing in the form of letters in the local press. The charges should certainly be thoroughly investigated and the truth of these matters made public. We must protest against the reiteration of the term "young girls." Nurses in general hospitals are now seldom admitted at an age to bring them under this description, and at and after twenty-four surely they may be called women. The committee at Newcastle will do wisely to put a stop once for all to the present accusations, and only a thorough and impartial inquiry into the nursing department can accomplish this. No doubt the question of the eligible age for probationers will receive due attention.

### DISCOUNTED CHARITY.

A governor's recent application for the reduction of fees charged for private nurses to employers who are subscribers to the institution received small encouragement at the Middlesex Hospital general court. The chairman reported that after careful deliberation the Weekly Board considered it inadvisable to grant the request. It is by no means unusual to find "discount off" demanded in charities. Not only are so many "subscriber's letters" expected by one donor of a certain sum, but when the recognised amount is collected from numerous sources the person who hands it in often considers himself entitled to personal repayment in the form of in or out patient letters equivalent to the whole sum raised. "People who refuse to give five shillings to a worthy object will cheerfully throw away ten or twenty in bazaar lotteries got up for the very same object," said a nurse to us recently.

### A NOTABLE HOME.

The annual meeting of St. Catherine's Home, Bradford, has taken place, great satisfaction being expressed by the numerous visitors at the appearance of the orderly household and at the pleasant and harmonious surroundings of the sick within its walls. "Hospital" as well as "Home" it should indeed be called, for the cases admitted require skilled treatment and trained nursing, and both are liberally provided by the founders and supporters of St. Catherine's. The chronic and incurable cases who spend the last days of their suffering lives in this valuable Home fully appreciate the kindly forethought which duly provides for their requirements.

### WOLVERHAMPTON.

A pleasant entertainment was given to the patients in the Wolverhampton workhouse infirmary the other day in the shape of a tea party, organised and carried out by a number of ladies. The Brabazon employment scheme has been received with much favour in this institution, and the sale of the work recently realised £21 12s., which will be spent in materials for next year. So long as these charming fancy articles are made by the male patients and by feeble or aged women only, the scheme deserves all praise, but it rests with the kindly visitors and workhouse officials to avoid giving this employment to the majority of female inmates, who should be exclusively employed on the needlework of the house itself.

### OUR CHRISTMAS COMPETITION.

Our next issue will contain the names of the prize-winners and also a list of all the contributions received for distribution in hospitals.

### SHORT ITEMS.

Nurse Repton has been appointed by the committee of the Stoke Parochial Nursing Association to succeed Nurse Ashton. — On January 17th Miss Mollett, Matron of Royal Hants Infirmary, Southampton, will read a paper on "Matrons Under the Poor-Law." Tickets, 1s. each, can be obtained from Mrs. Andrews, Secretary, 22, Cheyne Gardens, Chelsea.—On January 24th and 25th dramatic entertainments will take place at Queen's Gate Hall, South Kensington, in aid of the funds of the Nurses' Home of Rest at Brighton.—Mrs. Bishop has been elected an honorary member of the Pekin Oriental Society, the first lady who has found a place on its long roll of Oriental literary celebrities. —Over £80 was realized by the Doll Exhibition in aid of the Society for the Prevention of Cruelty to Children, recently held in Dublin.—The lectures on Nursing at the Wigan Mining and Technical School are well attended; the course is being given by Miss Alice Peck, late matron of Bootle Borough Hospital, and formerly matron of Longton Cottage Hospital.— A "Toy Service" at Lewisham Congregational Church resulted in 2,000 offerings for poor and needy children. —It is stated that a hospital which will cost 50,000 rupees is to be given to the Countess of Dufferin's Fund by a Parsee. The foundation-stone was recently laid by Lady Elgin at Karachi.—An anonymous donor has sent £500 to the Chelsea Hospital for Women, and similar sums have been promised from two other quarters.

### Christmas Fare Two Centuries Ago.

Who would not care to look into a middle-class household at Christmas time so long ago, and see how they kept their festival? Thanks to Mr. Wheatley's new edition of Pepys, recording for the first time many interesting details omitted by previous editors, we may now get a pretty complete picture of Yuletide in the Restoration times. Then, as now, the festivities lasted well over into the new year. Family gatherings were the rule, and elaborate displays of hospitality were considered a proper mark of worldly prosperity. The hours were not unlike those of our own day, and the amount of food and drink consumed on great occasions was prodigious. Breakfast, if company was invited, took place rather late. One such party, consisting of men only, Pepys records one New Year's Day, when he provided a barrel of oysters, a dish of neats' tongues, a dish of anchovies, wine of all sorts, and Northdown ale. The feast lasted with great merriment till about eleven, and we learn that one of the guests had that morning lost his only child: "Yet he was so civil to come, and was pretty merry." On Christmas Day service was held at ten or half-past, and again in the afternoon at three. Contrary to modern ways, it was the men whose attendance at church was considered indispensable; the ladies, unless very fashionable, were commonly detained at home by domestic affairs, and Pepys takes note of the "very great store of fine women" as an exceptional feature at Mr. Rawlinson's church. The most recklesss libertines and notorious sceptics were habitual churchgoers, and disgraceful scenes occasionally took place when the preacher took courage openly to rebuke any popular vice. One such occasion Pepys describes on Christmas Day, 1662, when at Whitehall Chapel a sermon was preached against the prevalent gambling mania, and every stricture of the learned divine was received with open laughter and scorn among the Court gallants present. Between the two services in church dinner was served at twelve, and a very substantial hot supper about seven concluded the meals of the day.

Christmas gifts had not yet come into fashion. Gentlemen in the country would, it is true, send turkeys, oysters, or brawn to their friends in town, and Evelyn seems to have made a practice of bestowing alms on Christmas Eve, and relieving the poor prisoners on Ludgate Hill; but the great time for presents was, after the French fashion, on New Year's Day. Thus we find Pepys on that day receiving a noble silver warming-pan from a friend; and his gifts to his wife are duly recorded—three pounds' worth of lace for a handkercher, or, more liberal still, a cabinet in walnut-tree wood, costing £11.

New clothes were a recognised feature of the festival. We learn how one Christmas morning "my brother Tom came to see my wife's new mantle put on which do please me very well," and again on the same anniversary how "my wife, poor wretch, sat undressed all day till ten at night altering and lacing of a noble petticoat." Pepys's own extravagances in the matter of dress are also noted since he made it a practice to pay all his bills at this season, and begin the new year as far as possible without a debt.

The bill of fare was far less stereotyped than is now the practice at this season in ordinary households. In the early days we find Pepys living in a garret, content with very simple dishes. On Christmas Day he is proud to sit down to a good shoulder of mutton and a chicken, and on New Year's Day is satisfied with the remains of a turkey dressed by his wife, who burns her hand in "doing of it." The following year he is distinctly unfortunate. He dines at home all alone, and "taking occasion from some fault in the meat to complain of my maid's sluttery, my wife and I fell out, and I to my chamber in a discontent." It satistisfactory to learn that "after dinner my wife comes up to me and all friends again," and that the evening concludes with an amicable stroll on the leads, but his New Year's Day is even more uncomfortable. He dines at noon with his cousin Thomas Pepys, a family party. "Here I first saw his second wife, which is a very respectful woman, but his dinner—a sorry, poor dinner for a man of his estate, there being nothing but ordinary meat in it." His supper at the house of another friend is worse still, the lady of the house being evidently no cook. They have "a calf's head carboned" (that is, cut crosswise and broiled), "but it was raw, we could not eat it; and a good hen. But she is such a slut I do not love her victuals." The next year his wife is ill, and, like a good husband, he dines at her bedside "with great content, having a mess of brave plum porridge and a roasted pullet for dinner, and I sent for a mince-pie abroad, my wife not being well to make any herself yet." It is difficult to trace the transition, probably very gradual, between the "plum porridge" of old tradition and the orthodox refection of to-day, but the mince pie played a very important part in the household. We find Mrs. Pepys just able to get about the following day, and devoting the whole of it to the making of Christmas pies, while she despatches her husband to Newgate Market to buy a bake pan at a cost of 15s. On subsequent festivals the making of the mince pies occasions great stir and ferment in the house. Christmas eve is commonly devoted to the business, and Mrs. Pepys, though now grown too grand a lady to do it all herself, has the maids to superintend, and keeps them up till four in the morning over this all important work. Turkeys and chines of beef figure conspicuously in the dinner parties at this season. "A hot swan pie" would be an interesting novelty now, and was even then a rather rare dish; Pepys observes that he did not eat any of it, and explains the fact by the "offence given him at the sight of his hostess's hand," who was also the cook. On the whole he enjoys his Christmas dinner best with his wife at home. His table improves with his fortune, and he can boast on January 1st, 1665, "At noon a good venison pasty and a turkey to ourselves without anybody so much as invited by us, a thing unusual for so small a family of my condition; but we did it and were very merry."

"Christmas gambols" of a very lively description filled up the programme after supper. These revels were occasionally of a more boisterous nature than Pepys could altogether approve, but his lady entered into them with the greatest zest, and once kept up the games in her own house till eight o'clock the following morning. Twelfth Night brought the Christmas gaieties to an end with the ancient ceremony of drawing the cake and choosing king and queen, attended by many quaint observances.

On the whole there is little to regret in the old times. Much louder mirth no doubt there was; very possible a far greater power of simple enjoyment. But children, the rich and the poor, are all conspicuous by their absence from any participation in the merry-making of Pepys and his friends, and who can bear to think in these days of a Christmas joy from which these are excluded?

### District Nursing in Holland.

It is by very slow degrees that our Dutch neighbours begin to realise that district nurses require special training. However, a progressive step was made at Zwolle in October when skilled nursing on the lines followed by the Queen's Jubilee Institute was inaugurated amongst the sick poor. Already the patients prove warmly appreciativs of what is done for them, and the doctors appear well content with the new departure, but the movement is still in its infancy, and cordial co-operation is required for successful organisation of this valuable work. The excellent series of articles contributed to The Hospital last winter on District Nursing, which are being reproduced in the Dutch nursing paper, will at any rate show the high standard and thorough training demanded from those who follow one of the most important branches of nursing.

### "Our Princess."

### H.R.H. THE PRINCESS OF WALES PRESIDENT OF THE ROYAL NATIONAL PENSION FUND.

When, a generation ago, the whole British nation rejoiced to welcome "the land's desire, Alexandra," there was a general aspiration that almost reached prediction that the beautiful girl, the gentle Princess, the exquisite bride, might be spared all knowledge of the griefs that darken human life. "May sorrow never dim those dove-like eyes," sang one poet, so popular in his own day and so utterly forgotten in ours as to make us speculate, not hopefully, on the fate which may, a generation hence, befal some of our own immortals. Only Thackeray, whose cynicism touched deeper things than the pathos of many of the poets, protested that without the touch of sorrow the life of the woman and the future queen would be incomplete, unfit for the task laid upon them. And the cynic was right.

### The Discipline of Pain.

A smiling, gracious, graceful personality, an ornament to the highest circles of national society, a fitting centre for a Court—all these the Princess of Wales would have been in any case; but it is not too rash to surmise that we owe the friend of the sick and suffering, the thoughtful and wise as well as benevolent lady, the sympathetic and tireless worker in every good cause who bears that title, to circumstances which in themselves were "not joyous but grievous." The Princess was yet but a young wife when she learned the long discipline of pain, and we find her from the sick-bed to which she was confined by acute rheumatism sending toys to a children's hospital. But this showed unselfishness rather than force of character; not in this act, kind and thoughtful as it was, shall we find the germ of that quiet womanly heroism which we have learned to associate with the Princess of Wales.

### Her Husband's Nurse.

But her own health had not been long re-established when the Prince's illness came. Our younger, more advanced and aggressive generation can hardly recollect that time, those weeks of breathless suspense for the whole empire, but in which the burden of watching and nursing fell on the shoulders of the Princess of Wales and the Princess Alice. It must be remembered that in those days trained nursing was by no means the developed and recognised profession that it is now. Noble women then were engaged in the noble work, but it was not the simple matter of course it is now to write or telegraph for a nurse to the nearest hospital or institution. The relatives of the sick, aided by their servants, did more, even in the highest rank, than is often dreamed of now. So by her husband's sick bed the Princess of Wales did her first nursing work. With what devotion it was done the outside world can just surmise, by noting in the newspapers of the time, so much less voluminous and personal than ours, how long a time had passed, how the crisis in the battle fought between Youth and Death had arrived, before she stole forth even to the church in Sandringham Park to join for a few minutes in the prayers which all England was putting up that day for the Prince's life.

### Doing Good by Stealth.

The first, the most direct and immediate results of that education in nursing which the Princess then had gained, were probably the visits paid to the dying groom who had caught the illness, destined for him to prove fatal, at the same time as the Prince. But from that day there has been visible a steadfast and ever-widening interest in all institutions and all persons that minister to the sick. We do not mean only formal State functions, to lay the foundation-stone or open a new wing of a hospital; there have been private visits not mentioned in the *Court Circular*, but remembered to-day in many a humble home. It is unfortunate that our English loyalty should force our Royal personages so often to do good by stealth. Thus, when the Princess of Wales and her daughters played at a concert given to the patients in one of our London hospitals, the fact of their intention had to be guarded like a State secret. If it had not, governors and subscribers, with their women-kind, would have forced or wheedled an entrance, and the patients, whose gratification the Princess sought, would have been thrust into the background. But these private visits and careful private inquiries have kept the Princess of Wales from extending her patronage to any of the very numerous institutions, dubiously philanthropic in origin and object, though loudly professing their high and useful aims, which yearly spring up around us. The common notion that Princes believe just what they are told does not hold good of either the Prince or Princess of Wales, who, by inspection and investigation, generally learn the true worth of the things which they honour with their approval.

### The President of the Pension Fund.

It is this knowledge which adds so much to the distinction conferred on the Royal National Pension Fund for Nurses by the Princess of Wales becoming its President. It is no formal, meaningless honour; it is the result of a firm conviction that the Fund, based on the union of help with self-help, is an acknowledged boon to a great number of hard-working and self-sacrificing women. Nor has the Princess given her name and done no more. The presentation of their certificates to the first thousand nurses who joined the Fund is just one of those things that sounds little but means much. It was done at the cost of some personal inconvenience, of an undeniable amount of fatigue which our agitators who talk of the "luxurious idleness" of the upper classes would do well to consider; and it was consciously intended and received as a token of appreciation of the prudence and common sense of those nurses who by joining the Fund had secured not only for themselves but for those who came after, the largest endowment ever offered to their profession. It was, in fact, the first testimonial ever offered to the business faculty in women. We do not speak rashly in ascribing so much to this gracious act, for the speech made by the Prince on his wife's behalf on that occasion showed such a full appreciation of the aims, methods, and results of the Pension Fund as must have surprised and enlightened not a few who heard it. In the following year Her Royal Highness also graciously presented the Second Thousand Nurses with their certificates, and the visit to Marlborough House will remain a most precious remembrance to each one privileged to attend the garden party.

### Sorrow and Bereavement.

Concerning this reception in 1890, which the *Daily News* declared to be the "prettiest sight of the season" there is one feature to which we now look back with sadness. Among those present with the Princess was the Duke of Clarence. Not a very long time was to pass before the Princess who had proved herself a nurse and the friend of nurses, was again to come face to face with suffering and death. We have almost forgotten, in face of the sudden bereavement that followed, how it was the Duke of York who first was taken ill, in that fatal month of December which had witnessed the death of the Prince Consort and the Princess Alice, and had seen the Prince of Wales in close combat with death. But the Duke of York, with his strong constitution developed by his sailor life, conquered the danger that threatened him, and after weeks of anxiety the royal household were able to go to Sandringham for Christmas. And there, the expected dart having been averted, the unexpected fell. In January the

OUR PRINCESS.

Alexandra

President of the Royal National Pension Fund for Nurses.

*By permission, from a photograph by Van der Weyde, Regent Street, London, W.*

Duke of Clarence was taken ill. It was a short sharp illness, and the Princess who had just brought one son back from the brink of the grave could not save the other. The Duke of Clarence died, and how much of his mother's heart went with him to the grave only mothers who have been bereaved can know.

The Chastening of the Lord.

For our Princess has not been the same since; while there has been no selfish isolation in her grief, no refusal to perform the duties of her station, it has been clear that the more frivolous forms of entertainment have less attraction for her than ever. The marriage of the Duke of York has given the nation another popular princess who may fitly take upon her young shoulders the burden of the lighter side of social life, relieving the Princess of Wales as she, thirty years ago, relieved the Queen. But to the suffering and the sorrowful the Princess's heart goes out even more readily than before. Witness, if witness were needed, that hurried journey across Europe to the death-bed of the Czar, undertaken too late, indeed, to be of help to him, but not too late to be of inestimable comfort to the widowed Czarina, whose strength, already tried by long years of gilded terror, might well have given way under her great loss. Even those who are most loth to see any good in Royal personages are compelled to admit that the presence and conduct of the Prince and Princess of Wales in Russia at this time have been of the greatest value, not only in comforting their kinsfolk, but in bringing about a kindlier feeling and a better understanding between two of the greatest nations of the world—nations whom circumstances have conspired to place for a long time in jealous opposition to each other.

A Silent Force.

We have been perhaps inclined to take too little heed of the influence of the Princess of Wales. It has been silent, unobtrusive, womanly. In the midst of a shrieking and hysterical generation she has preserved a gracious and dignified reticence. No selfish and aggressive cause, however loudly trumpeted, has won her approval; no combination of vanity and self-will, posing under an imposing name, can boast that she has upheld them. But when our children shall estimate the worth of the prominent men and women of our time, they will see how a stream of blessing, beginning in the fountain head of home—home joys and home sorrows—has flowed forth to benefit and enrich the land of her adoption from the life of Alexandra, Princess of Wales.

### How, When, and Where Nurses Spend Christmas.

By a Nurse.

"Nothing can be so mean
Which with this tincture (for Thy sake)
Will not grow bright and clean. . . ."
*George Herbert.*

"God bless you and all good ladies who have discovered that human beings have bodies as well as souls, and that the state of the soul too often depends on that of the body. . . ." —*Charles Kingsley.*

### HOW?

A First Christmas.

"How did you like the first Christmas Day you ever spent in hospital?" said a probationer, looking wistfully into my face one Eve. I was very busy putting the patients' presents in orderly array to facilitate their distribution by the night nurse in the early dawn, and I pinned the label on to the last of the queer-shaped parcels before answering.

"Not so well as succeeding ones. I was a little bit homesick, and oh! so tired," I said. The probationer smiled, "Yes, you don't forget that new-comers get easily tired," she remarked, "but I can't picture you dull or home-sick."

She still looked rather wistful, so I lingered to add, "Well, I remember we had some carol singing on Christmas evening, and one of the tunes upset my nerves, and I wanted to cry! As there was little chance in the ward of doing so unobserved, I slipped away to the ward kitchen to wipe my eyes comfortably, you know! But, alas! it was already occupied by another probationer, and her red eyes showed she had come there for the same purpose. So we consoled each other, had a hearty laugh over our 'sentiment,' and soon forgot it in making things pleasant for the patients. After all, home-sickness is often a symptom of over-fatigue, isn't it?"

In Hospital.

There is not much monotony in hospital Christmases. Plans for patients and schemes of decoration fill up every moment that can be spared from the actual work of nursing. Even the feeblest of patients evince some interest in the approach of the festival, and doctors are willing to lay aside professional gravity when old patients offer seasonab'e greetings, and pert juveniles suggest Christmas boxes.

After all the character given to Christmas Day in the wards largely depends on the heads of the nursing department. If they are unselfish and sympathetic, both workers and patients are sure of "a good time" in every sense of the word. Officials may lay down strict rules as to the character of the entertainment permitted, and they may be generous or mean as regards practical assistance; but it is to the nurses that the patients turn instinctively. They appreciate the kind interest of strangers, but it is only to the nurses that they speak in free criticism or praise. After all, the hospital is a very nice place in which to spend the festival. Even those to whom "looking back" is made sad by old memories, the attempt to shed a little temporary brightness on the dreary lives of others, makes "not such a bad Christmas after all."

Out of Hospital.

Perhaps "private" is the most trying branch of our work to a nurse at this season. "The special" who spends her day beside the bed of a bad case in hospital remains in touch with her fellow workers, who show kindly consideration for one who is thus kept apart by duty. The private nurse, however, often feels very much alone in a strange household, and it needs all the cheerful patience she is possessed of to prevent her from showing this and adding, however little, to the heavy burden of sickness which oppresses the home-circle of which she is a temporary member.

### WHEN?

"Why, on December 25th, of course." Well, not exactly! It gets "kept" otherwise sometimes. What about the night nurse, for instance? Often after the twelve strokes of midnight have sounded solemnly in the silence many a nurse remembers occasions when "A happy Christmas to you, nurse," from some wakeful patient has been the first reminder of the new day. There is something very touching in this greeting, bandied about from one patient to another in friendly tones, as each sick man or woman rouses from slumber. In large hospitals it is frequently impossible for Christmas dinners to be served for staff and patients on the actual day, so the festivities are spread over several dates. "A very bad case" too may cause the postponement of the concert or other amusement till a later day. "Patients first and pleasure after" should be the invariable if unspoken rule.

### WHERE?

Abroad.

"Do write again soon, home news is so welcome," constantly recurs in the letters of absent nurses, "we thought of you at Christmas, and wished we could see what you were doing." These old nurse-friends of ours have memory to help them in their fancies, but we at home can only guess at their strange surroundings.

In India, Hong Kong, Australia, Ceylon, Egypt, Italy,

America, Canada, St. Helena, and in Africa our fellow-workers are proving the value of skilled nursing to many a white and coloured patient. Army nursing sisters, missionaries, deaconesses, and lay nurses are alike engaged in the never-ending conflict with disease and death. Victories are won, glorious, bloodless fights, of which no record exists save in the grateful hearts of those whose dear ones have come back from the very gates of death. Many a nurse's sole reward lies in the doctor's quiet "Your patient does you credit, nurse."

At Home.

Some of the hospitals seem to antedate Christmas in their festivities. The concert at St. Marylebone Infirmary took place last week, and the fine blocks of buildings were traversed by numerous guests, and an enjoyable evening resulted.

Two evenings in the same week were devoted by the women medical students at the Royal Free Hospital in Gray's Inn Road to entertaining the patients. Musical and other performances took place in the wards for the benefit of those who were not well enough to attend the entertainment in the board-room enjoyed by convalescents and nurses. The students also kindly provided presents for the patients. The excellent programme of amusements, which has generally occupied two or three evenings at Charing Cross Hospital, has been discontinued in favour of festivities in the wards themselves; gifts for the patients and elaborate teas being, of course, essential features of Christmas Day.

A pleasant concert took place last week at the City of London Hospital for Diseases of the Chest, at Victoria Park. It was arranged by the senior assistant physician, Dr. T. Glover Lyon, and was universally appreciated.

The present students at the School of Medicine for Women in Handel Street, W.C., hospitably entertained a number of qualified lady doctors and past students on 17th inst. The gathering was exceedingly pleasant and the large rooms were well filled. The verandah was picturesque with coloured lanterns, whilst graceful decorations of evergreens gave a festival air to the interior of the school. In the lecture room—Tableaux, a Farce and a Topical Dialogue successively won much appreciation. There was some very good music, including songs by Mrs. Edith Dick, whose charming composition, "Spring is Here," was received with well-merited applause for its own merits as well as for its artistic rendering. Miss Gethen, formerly Sister Queen, recited some of her original "stories" to a most appreciative audience.

At the London Hospital entertainments of various kind are arranged at Christmas in each group of wards, many of the amusements being organised by students and other friends of the hospital. Daintily arranged tea tables and seasonable decorations and gifts combine to make the Christmas spent in hospital a pleasant memory to many a poor East-ender.

Presents for each patient, Christmas fare, a beautifully decorated little chapel, and a sacred concert on the following Sunday, are the characteristics of the season at King's College.

At St. Thomas's, the carols sung by the Nightingale probationers in each ward have long been noteworthy. It takes a whole evening for this seasonable programme to be carried out, the fine wards, gaily decorated and lit up, forming a fine perspective study. It has been rumoured that the concerts usually given in the wards during Christmas week, which have always been particularly good ones, will be discontinued this year.

A gigantic Christmas tree makes its appearance annually in the board-room of Middlesex Hospital, loaded and surrounded by presents, all patients who are sufficiently convalescent being invited to come down on Christmas Day to the distribution of gifts. The Hospital Musical Society gives occasional concerts in the course of the year, which are much appreciated by the patients and staff.

At the Children's Hospital, Great Ormond Street, the entertainment for the little patients is fixed for December 27th, when visitors are invited to the wards from half-past two to five; tea being provided from five to six.

An abundance of good cheer is in course of preparation for the patients and staff of the Chelsea Infirmary. Prime joints are on order at the butchers, and plum puddings and mince-meat from a recipe of which the cook is justly proud are all ready for use in the larder. The wards will be decorated as usual, but considerable mystery prevails as to what shall be the distinquishing colour of each. Arrangements are being made for the entertainment of the patients in their respective wards, and a pleasant and harmonious Christmastide is anticipated.

A Christmas tree and sale of work in aid of the Nurses' Home, Howards' Road, was held in the Public Hall, Canning Town, on Wednesday, December 19th. It was opened at half-past two by the Countess of Warwick. Recitations, waxworks, and music were amongst the entertainments provided.

A resolution that £5 be spent in providing toys for the children in the South Dublin Union was unanimously passed by the Guardians at their weekly meeting on the 13th inst. The usual Christmas allowances will be issued and school holidays given, but none of the usual gatherings will be held in the workhouse this year on account of the continued prevalence of small-pox. Estimates as to the cost of increased temporary accommodation, both for nurses and patients, have been invited by the Guardians in case the progress of the epidemic should render its provision necessary.

At Guy's Hospital the pleasures of Christmas are spread over the week ending only with the first day of the new Year. Entertainments are provided in turn in the various wards, the students taking a great deal of trouble in amusing the patients.

At the North London or University College Hospital the annual entertainment will take place on Wednesday, January 2nd, between three and eight.

The Christmas Entertainment for the East London Hospital for Children at Shadwell is fixed for Saturday, December 29th. Tea will be served to the children at 3 p.m.

On Monday evening, December 17th, the large dining hall at the Workhouse at Fir Vale, Sheffield, was crowded with inmates to witness the performance of an operetta in two acts, by the nurses of the Workhouse Infirmary. Amongst the visitors present were Messrs. Hoyland and Senior, Vice-chairmen of the Board of Guardians, P. H. Ashberry, A. Craven, W. D. and Mrs. Forsdike, J. C. Stott, Dr. Collier, Mr. Waters, workhouse master; Mrs. Ward, matron; Miss Ward, Oldham General Infirmary; Miss Thomlinson, infirmary superintendent; and Mr. and Mrs. Offen, superintendent and matron of the children's homes. The entertainment at this workhouse has become an annual event, and this year it passed off as successfully as usual. At the close of the dramatic performance, by the kind permission of the Guardians, the nurses and guests had a dance in the entrance hall of the workhouse.

With the Incurable Children.

Such a terrible word "incurable!" seems that it is spoken hesitatingly of elderly persons, and to use it for a little creature just entering on life seems a hideous mockery. Yet there is nothing "hideous" about the small people at the home and hospital at 2, Maida Vale. "Do they like Christmas?" was asked of the matron. She smiled as she answered, "They anticipate it for three months, and look back on it for six." Of course presents and Christmas fare form considerable items, but the fanciful, precarious appetites of chronic invalids are soon satisfied. On cards and letters they simply dote, the more they get the better they are pleased, and every stray penny is hoarded by these quaint little patients to pay postage, the aim of all their communications being "to get an answer back."

### Help the Nurses to Help the Sick.

In this homely and appropriate phrase do we each year suggest to our readers a few of the many societies which will "gratefully receive and acknowledge" donations large or small. We usually find our appeals are approved of and we trust this present Christmas will be noted for increased generosity on the part of all who are able to enjoy "the luxury of giving"—

"This is true liberty when free-born men
Having to advise the public may speak free;
Which he who can or will, deserves high praise;
Who neither can nor will, may hold his peace;
What can be juster in a state like this?"
"*Euripides," translation by Milton.*

**The Royal National Pension Fund for Nurses.** Offices, 28, Finsbury Pavement, London, E.C.—The Fund has had another very satisfactory year, the business during 1894 showing a considerable increase over 1893. The invested funds are now just upon £200,000, and when one looks back and sees that at the end of 1890 this total stood at £80,000, the rapid development of the Fund certainly strikes one as being most remarkable. The fact that each year the number of new policyholders is greater than during the previous twelve months clearly shows that nurses fully appreciate the benefits to be derived from joining the Fund. During 1894 over £700 has been distributed in sick pay. The vacancy on the council caused by the death of Mr. Clifford Wigram, late Deputy-Governor of the Bank of England, has been filled by Mr. Herbert C. Gibbs, of the firm of Anthony Gibbs and Sons, one of the most prominent City houses. At the last election for annuitants and policyholders' representatives, Miss Wedgwood, Matron of the Royal Free Hospital, and Miss Arabella Wood, of the Nurses' Co-operation, were added to the already very strong list of ladies connected with the management of the Fund. The position of the Fund has been materially strength ened by a successful action which the council found it necessary to bring in vindication of the rates charged for pensions, and which very conclusively proved that in the Fund nurses are able to secure substantial and exceptional advantages. During the year the offices have been moved from 8, King Street, Cheapside, to the above address, where all further particulars can be obtained by writing or applying personally to the Secretary.

**The Junius S. Morgan Benevolent Fund,** which is an auxiliary to the above fund for nurses, has an excellent record, for it has already dealt with the cases of nurses who, without this aid, would have been destitute. The business of the Royal National Pension Fundis admirably managed by men of knowledge and experience, the work of the Benevolent Branch is done by volunteers, under the supervision of an influential committee, of which Lady Rothschild is President, and is most efficiently carried out by the Hon. Secretary, Miss R. Pritchard, who devotes time and care to the investigation of claims and the relief of urgent cases. The report of the fund shows excellence of its organisation and management. About 25 nurses are now life pensioners, and no nurse is permitted to leave the Pension Fund until every effort has been made to afford her assistance to tide over her difficulties. Fresh subscriptions are now required to extend help to those outside the Fund.

**Queen Victoria's Jubilee Institute for Nurses.** Offices at St. Katharine's Hospital, Regent's Park.—The Institute trains probationers and supplies women trained in district as well as hospital nursing, to the sick poor in their own homes. Applications for information should be addressed to Miss Peter, the Inspector. Scotch, Irish, and Welsh branches also exist, and numerous rural associations have affiliated themselves with the Institute.

**Metropolitan and National Nursing Association, Bloomsbury Square, W.C.**—This is the Central Training Home of the Queen's nurses, where instruction in district nursing, lectures, and other educational advantages await probationers who have gone through a course of hospital training. Miss Amy Hughes is Superintendent of the Central Home.

**Queen Charlotte's Lying-in Hospital.** — This hospital was established in 1752. Her Majesty the Queen is patron, and their Royal Highnesses the Princess of Wales and the Duchess of York are vice-patrons. The committee earnestly plead for support to enable them to clear off the deficit of £1,300 on last year's accounts. Contributions sent to the bankers, Messrs. Cocks, Biddulph, and Co., 43, Charing Cross, or to the secretary, at the hospital, will be most thankfully acknowledged.

**The Hampstead Hospital** (The Hampstead Home Hospital and Nursing Institute) has during the past year altered its constitution, the general wards being now free to the poor of Hampstead, Highgate, and adjoining neighbourhoods. This will meet a want long felt of a general local hospital. Commencing with one house, it now comprises a block of four, with a separate house, connected by a covered way, for the private nursing staff. During the past year nearly 250 patients have been treated, in addition to a large number of emergency cases. This institution deserves more local support. Hon. Secretary, Mr. R. A. Owthwaite.

**National Health Society.**—The basis of the whole system of instruction which this most useful society comprises, may be said to lie in the proverb that "Prevention is better than cure." It goes straight to the root of the matter, as its aim is to diffuse by all possible means sanitary knowledge of every kind, comprising home nursing, rearing of infants, prevention of the spread of infectious diseases, and, by no means least important, lectures on sanitary reform in food and cookery. The society gives courses of lectures on the various subjects which affect health, not only in London and its vicinity, but even so far afield as Glasgow and Aberdeen. We wish this useful society all success in its work. Secretary, Miss Lankester, 53, Berners Street, W.C.

**The Invalid Children's Aid Association.**—This ideal charity has its offices at 18, Buckingham Street, Strand. The object of the association is to help invalid and crippled children in their own homes. A system of regular visiting is organised, and the ladies who volunteer for this work find out what is most needed by the little people, and endeavour to supply it. Spinal carriages, perambulators, surgical instruments, and bed linen are lent, and clothes, toys, picture books, &c., are given. Tickets for seaside and country convalescent homes are distributed—in fact, much useful work is unob trusively done by this association. Funds are greatly needed by the committee to enable them to carry on their work. Subscriptions are gladly received by the Hon. Secretary, Mr. Allen Graham.

**Workhouse Infirmary Nursing Association.** Offices, 6, Adam Street, Strand.—Much progress is being made in introducing an improved system of nursing into workhouse infirmaries, but much yet remains to be done. The association has trained 250 probationers, and 130 nurses are now at work. In many cases the matron and staff are supplied to country union infirmaries. Increased financial support is urgently pleaded for, to extend the work of training proba tioners, to which purpose the greater part of the funds are applied. The objects of the association are: To raise the standard of public opinion on the whole question of workhouse nursing; to secure the appointment of trained ladies as matrons in all separate infirmaries; and to train and supply nurses to workhouse infirmaries in London and the provinces. H.R.H. Princess Mary Adelaide of Teck is President of the association, and subscriptions will be gladly received by the

Hon. Secretary, Miss Wilson, 13, Barkston Mansions, S.W.; or they can be paid in to the account of the W.I.N.A., at Lloyd's Bank, 215, Strand, London.

**Zenana, Bible, and Medical Mission,** 2, Adelphi Terrace, W.C.—This society has a threefold object, addressing itself to the spiritual, mental, and bodily needs of the women of India. The great desire of the mission at the present time is for friends of the work to subscribe £25,000 to enable the Council to consolidate the Zenana school and medical work of this society. Hon. Finance Secretary, Mr. W. T. Paton; General Secretary, Rev. A. R. Cavalier.

**East-End Mothers' Home,** 396, Commercial Road, London, E.—Twelve beds are available in this, the only maternity hospital in the densely populated districts of East London. In addition to the home, giving shelter and comfort gratuitously to poor working women, the out-patients' department supplies skilled attendance to mothers in their own homes at a fee of 3s. 6d. for each confinement. There were 196 in and 255 out-patients in 1893. Help is most earnestly requested for maintenance and extension. Bankers, London and Westminster Banking Co. (Limited), 1, St. James's Square. Mr. Arthur W. Lacey, Secretary.

**East London Nursing Society.**—The sick poor in East London have benefited by the work of this society during the last 26 years. Its labours extend over 33 parishes which have a total population of about 313,000. Hon. Treasurer, Mr. Percy Wigram; Secretary, Mr. A. W. Lacy, 49, Philpot Street, Commercial Road, E.

**The Ladies' Sanitary Society.**—This society, deserving of every encouragement, was founded in 1859, and at its first meeting Charles Kingsley called it "one of the noblest, most right-minded, straightforward, and practical conceptions that he had come across for some years." Particulars of the work of the society can be obtained from Miss Rose Adams, the Secretary, 22, Berners Street, London.

**St. Marylebone Female Protection Society,** 157, 159, Marylebone Road, N.W.—This institution is one of the oldest charities in London, and is engaged in rescuing young women who have been led astray. To send such women to the workhouse means further degradation, and therefore this society steps in. Highly gratifying results have attended its operations, as may be seen by the annual reports. Contributions are much needed and should be sent to George Scudamore, Esq., secretary.

**Royal Sea Bathing Infirmary for Scrofula,** founded at Margate 1791.—This unique and valuable institution is so badly supported that many of the fine wards have been reluctantly closed. It is grievous to think of the empty beds (more than half the total number) in those noble rooms, when so many patients might there be restored to health and usefulness. It is the only hospital in England set apart exclusively for scrofula, and the cures which result from a lengthened stay are indeed remarkable. We are glad to hear that wards containing ten beds have been reopened during this year. Of all the institutions which call for liberal support from Englishmen and women this fine hospital is one of the most worthy. All information will be gladly given by the Secretary, Mr. Arthur Peirce, 30, Charing Cross.

**Irish Distressed Ladies' Fund,** 17, North Audley Street, W. Patron, the Queen.—Relief is given independently of any question of politics or religion; employment is found, when possible, for those able to work; pecuniary help is given to the aged and infirm; and the education of children is paid for. Secretary, General W. M. Lees.

**Samaritan Societies.**—If well administered these societies are of infinite value in extending the benefits of hospital treatment. Through their agency patients are sent to convalescent homes; supplied with surgical instrument or warm clothes; and so the work commenced in the hospital is carried on outside. Some such society should be attached to every large hospital.

**Auxiliary Assistance.**—An association or guild for supplying the sick poor with invalid comforts and nourishment, should be an adjunct of every district nursing institution. The places where they already exist are increasing annually, and the value of this practical department cannot be over estimated, and is therefore deserving of substantial support.

### Christmas on an Australian Fruit Farm.

My brother invited me to spend last Christmas with him at his fruit farm on the Murray River. I had been nursing one or two rather trying cases in Adelaide, and was glad enough to leave the hot glaring town for a few weeks. I was to keep house for my brother and his friend, and they promised me all sorts of Christmas gaiety.

After a tedious coach journey along the Murray and the Darling I at last arrived at Renmark, where the twenty acre fruit farm was situated. Although pretty well used to roughing it, I was somewhat staggered to find that his "house" consisted of two wooden rooms. His friend had put up a tent at one side to serve as his bed-room, for they had given up the sleeping-room to me, and my brother contemplated sleeping on what they euphemistically called the "sofa," in the sitting-room. They had been very busy making the place smart and tidy in my honour, and both boys (for they were little more) surveyed the effect of their handiwork with evident satisfaction.

I soon "shook down," and a merry trio we were; we had several neighbours, some just fresh from England, and all gave me the heartiest of welcomes, for a woman was a rarity among them, and lucky was the man who had one to cook and "do" for him!

Christmas Day passed happily, for everyone did his best to be good to his neighbour; we danced a little, though it was too hot for this to be really enjoyable. We had a large dinner party at one of the oldest settler's houses, and our hostess proudly produced a plum pudding on fire, for she was determined to be orthodox, and we applauded her principles, but felt more inclined for strawberries and cream than for the flaming pudding.

We strove to be extra merry because, I think, we were afraid of betraying how horribly homesick we were; most of us had inconvenient lumps in our throats when the time-honoured toast of "Friends at Home" was drunk.

Christmas over the colony set to work again, and I was amazed at the amount the young men accomplished. They started out in the early morning with axe, shovel, and mattock to dig holes or clear scrub, and only returned at dark. No eight hours' day for them! They were mostly preparing for the future by planting lemons, vines, and apricots. Many had been drawn to the colony by the glowing accounts circulated at home of the £540 realised by one year's crop of apricots off nine acres of land, and some of the new arrivals had not taken into consideration that even "out there" newly-planted trees do not immediately bear fruit.

It was a pleasant, sociable colony, and primitive though it was, it seemed the height of civilisation to one who, like my brother, had been boundary-riding "out back" for a year. And as for me, I returned refreshed and invigorated, in spite of the long journey, to my own special calling of sick nursing.

E. G.

### Notes and Queries.

### Queries.

(52) *Housekeeping.*—Can you recommend me a book on hospital management and housekeeping?—*Nurse H.*

(53) *Training.*—How shall I get information about children's hospitals if I am too young for general hospitals?—*E. M. D.*

(54) *Maternity Training.*—Is there any hospital in Bradford where I could get this training?—*Monthly.*

### Answers.

(52) *Housekeeping* (*Nurse H.*)—If you are a trained nurse you had better seek a post as home sister or assistant matron in a hospital or infirmary. It is only by practical work under a really good matron that you can obtain the knowledge you seek.

(53) *Training* (*E. M. D.*)—Write to the matrons of several children's hospitals, and ask for their rules and conditions of training. You will find a list of these institutions in Burdett's "Hospital Annual," published by the Scientific Press, 428, Strand, London. You might also write to the Hon. Secretary, Workhouse Infirmary Nursing Association, 6, Adam Street, Strand, as regards the training granted by that admirable society.

(54) *Maternity Training* (Monthly).—There is no maternity hospital in Bradford. Would not Manchester or Liverpool suit you?

### Christmas Books.

### The Children's Shelf.

Nothing marks the difference in manners and thought which a hundred years has brought about more forcibly than a comparison between the children's books published in the present day and those prepared for their delectation at the beginning of the century. Here on the one side lies a pile of copiously illustrated, gaily bound volumes in the stout modern octavo which refuses to go into any pocket and makes a dashing, not to say glaring, show on the bookshelf. On the other side lie the tiny duodecimos in insignificant marbled covers which our grandmothers delighted in. Quaint wood-cuts of tiny boys in trousers and tailcoats and young ladies in high mob caps adorn them, nor does the excellence of the modern processes out-weigh the pleasant humour and observation revealed in these by J. Bewick. The binding both in taste and execution is all to the advantage of the old-time workman. How many of these showy-looking books of to-day could stand a hundred years of thumbing and knocking about and turn up at the end merely worn at the edges and discoloured a little, but with no leaf loose, no stitch undone? The stories themselves leave, it must be confessed, a good deal to be desired. These were the times immediately preceding Miss Edgeworth, and an immense deal of moral padding of the kind which children never can have endured, was considered the right thing for them. The very titles are discouraging. "The Blossoms of Morality Intended for the Amusement and Instruction of Young Ladies and Gentlemen" has no very promising sound, and "The Happy Effects of Sunday Schools on the Morals of the Rising Generation," where the village children confide to each other "theremust be something vastly pretty in being able to read the Testament," can hardly have proved amusing in that day, however curiously instructive in this. "The Interesting History of a Baronet's Daughter" is more stimulating, even though it is "intended as an instructive lesson for youth," and the promise of the title is actually borne out in the sequel where "Miss," as she is called throughout, falls in with "an elegant and fascinating young nobleman" and marries him at the mature age of fourteen. But to return to the year 1894; "Be cautious, my youthful readers, how you place too great a confidence in the possession of wealth and beauty, but fortify your minds with religion and virtue and a proper knowledge of the useful sciences." Something very like this translated into easy colloquialism is still the burden of "books for the young."

Two Girls.

"Two Girls,"* by Amy E. Blanchard, is an American story intended for what our old books would have called "female perusal." There is no novelty either in incident or treatment, and the liveliness of the book depends rather more on practical jokes and American slang than some folks would think quite in the best taste. Still the tendency is for good, the sentiments irreproachable, and the sprinkling of sentiment just sufficiently diluted to prove not unwholesome for the maids in their early teens for whom it is designed.

L. T. Meade understands better than most writers the sort of thing which girls really like, and "Red Rose and Tiger Lily"† is sure of instant popularity. Plenty of incident and movement worked out among a really charming group of girls, real troubles bravely met, and a spice of romantic improbability, setting everything straight, make up a very stirring volume. The happiest touches of all are in the persons of the tiny children, Nell and Boris, and the scene in the midst of the fancy ball, where these two, dressed as Fairy and Brownie, catch sight of their ruined father standing outside in the moonlight and run to comfort him is very finely conceived. Boris, deciding "that even if disappointment were in store he could all the rest of his life reflect that he had sat up late and eaten lobster salad for supper," or Boris, rubbing himself against his father's knee, and pleading, "We want you dreadfully, *dreadfully* in the house," or again, feverishly anxious at the thought of leaving the old home because of the big rabbit's feelings, and "awful feared it will kill him if he leaves his corner of the hutch," is uniformly boylike and charming.

The word "adventure" in the title-page and a frontispiece representing a fall down a precipice will be quite sufficient to attract the boy world, if left to choose for themselves, to "Stirring Tales of Colonial Adventure,"‡ by Skipp Borlase. They will not be disappointed in the expectation of finding plenty of fighting, brave deeds of a rough and tumble character and hair-breadth escapes. Although there is nothing actually objectionable, the adventures are not exactly carried on in kid gloves, and this is not one of those "boys' books" which a discriminating friend would select as likely to serve equally well for the girl portion of the family.

The Christmas volume of "Little Folks"§ makes a very

* Two Girls. By Amy E. Blanchard. (G. Newnes, Limited. Price 3s. 6d.) The Illustrations by Ida Waugh, one of which we reproduce, are graceful and pretty.

† Red Rose and Tiger Lily. By L. T. Meade. (Cassell and Co., Limited. Price 3s. 6d.)

‡ Stirring Tales of Colonial Adventure. By Skipp Borlase. (Frederick Warne and Co.)

§ 'Little Folks' Christmas Volume, 1894. (Cassell and Co., Limited Price 3s. 6d.)

pretty present for the younger children. As usual, the illustrations are excellent, and the letterpress, without one dull page, is full of varied and interesting matter. A good novelty is the "Pages for Very Little Folk," which are far more cleverly adapted to promote "Reading Without Tears" than even that time-honoured friend of our infancy. What would the "assiduously cultured young persons of both sexes" at the beginning of this century have said to such a volume as this?

### Victories over Pain.

Philosophers have about given up writing long treatises in order to teach people to bear their woes. They have discovered, in the course of some centuries, that nobody can be reasoned into consolation, and are even approaching the perception that pain is best borne when it is forgotten, and can only be forgotten when something better is put in its place. As long as the sick man lies "honing and moaning to himself"; as long as "his bowels are melted within him, to think what he suffers," it is no use preaching to him about the duty of cheerful resignation. "He has put on the strong armour of sickness; he is wrapped in the callous hide of suffering." But lift him out of himself, give him something else to think about, rouse him to smile, or better still to laughter, and the whole aspect of affairs is changed directly. The world no longer seems a vast cruel machine aiming at his destruction, nor the next paroxysm of pain a personal injury to be resented against mankind. It is the special value of Christmas time in the hospitals that it calls upon nurses and patients alike to throw aside private griefs and join in an effort at common gladness. There will be many aching hearts among those willing workers who decorate the wards; many tears, evoked by memories of the happy past, will be choked back under cover of merry chatter, many sighs changed in their birth to smiles, lest any shadow should dim the brightness of the Christmas peace. And many who have grown to dread the thought of the old home festival will look back with wonder after it has passed at the real joy and consolation wrought out of this vigorous discipline which has lifted them above private sorrow.

The Black Dwarf.

The appearance of the fifth volume of the Dryburgh Edition of the Waverley Novels* recalls perhaps the most forcible example of this contempt of pain which literary history can afford. For one of the Tales of My Landlord which it contains, viz., "The Legend of Montrose," with all its verve, movement, and wealth of humour, was dictated by Scott through such agonies of his inveterate malady, cramp in the stomach, as might, one would think, have made it impossible to think of anything but his own misery. "The affectionate Laidlaw beseeching him to stop dictating when his audible suffering filled every pause," "Nay, Willie," he answered, "only see that the doors are fast. I would fain keep all the cry as well as all the wool to ourselves; but as to giving over work that can only be when I am in woollen." Though he often turned himself on his pillow with a groan of torment, he usually continued the sentence in the same breath. But when dialogue of peculiar animation was in progress, spirit seemed to triumph altogether over matter — he arose from his couch and walked up and down the room, raising and lowering his voice, and, as it were, acting the part." The Dryburgh Edition should be welcome in many households by reason of its convenient size, excellent print, and moderate price. One of the very spirited illustrations, drawn by Walter Paget for "The Black Dwarf," we reproduce.

### Books of Devotion.

When the Red Cross Knight, weary with much conflict, sought a refuge under the guidance of Mercy from continued strife, she guided him along the painful way—

> Forth to an hill, that was both steep and high,
> On top whereof a sacred chapel was,
> And eke a little hermitage thereby,
> Wherein an aged man did lie,
> That night and day said his devotion
> No other worldly business did apply;
> His name was Heavenly Contemplation,
> Of God and goodness was his meditation.

The life of the world, and the life of religion or the cloister

* Waverley Novels (Dryburgh Edition). Vol. v., price 3s. 6d. (Adam and Charles Black.)

were thus sharply sundered. The warrior might find consolation from the monk, might share his solitude for a period, and return to his work a better man, but that he should live in the world a life equal in religious aim and purity to that of the "reilgious" man himself was not contemplated by either. The complex duties of court and camp were in direct opposition only too commonly to the imitation of Christ, and he only could be held to attain true heavenly contemplation of whom it could be said, "Ne other worldly business did apply." This feature of the middle ages it is well to bear in mind in the study of the most beautiful and enduring of all the religious works to which they gave birth.

A new edition of "The Imitation of Christ"* is rendered more attractive to a very large circle of readers by the introduction prefixed from the pen of Dr. Farrar. But it is very easy to see that the editor is not altogether in sympathy with his author. He is repelled, one might almost say annoyed, at the monastic spirit of the work, and although in the course of his able discussion as to its probable author he takes note of the miserable conditions of public morality under which it was composed, he comes perilously near blaming the writer for the existence of the vices which he abhorred. It is to the last degree misleading to accuse him of holding the conviction "that life is miserable, society incurable, the intellect dangerous, and the world hopeless." He held, indeed, no rose-coloured views of his times, but the secular writers go a long way further in their denunciations of the whole fabric of society as then constituted. The special doctrine, however, of the author of the Imitation, which men of every age since and every class have found to stimulate them to action, is that society can be regenerated, not by eloquence of preaching, still less by the "glorious refreshment we may innocently derive from things beautiful alike in nature, in literature, and in art," but solely by the self-dedication of the individual, united to God. "Love not pleasure; love God; this is the everlasting yea wherein all contradiction is solved."

There are many busy people—

> Who carry music in their heart
> Through dusky lane and wrangling mart,
> Plying their daily task with busier feet,
> Because their secret souls a holy strain repeat.

A little volume of meditations called "Searchings in the Silence," † by Dr. Matheson, is eminently suited for those whose little hermitage has to be framed out of odd moments or stolen from much-needed rest. Each reading occupies about two pages only, but is pregnant in this small compass with suggestion and consolation. For reading to the sick and sorrowful few better books could be recommended. Without any straining at effect there is just enough freshness of treatment and originality to render Dr. Matheson's thoughts acceptable even to those somewhat foredone with exhortations. The following brief extract is a specimen of its bracing tendency: "There are times when grief itself must be sacrificial—self-concealing. There are seasons in which sorrow has to put on the appearance of brightness. And there is no burden in the world like to that. It is hard enough to suffer; it is harder still to hide suffering; but it is hardest of all to simulate joy in suffering. . . . To keep up under a load lest a brother should be borne down, to anoint the face in sorrow that a comrade in pain may be relieved—it is to wear the smile of God."

Messrs. Burns and Oates issue a handy little "Devotional Library,"‡ in cloth case, containing the New Testament from the "Vulgate," the Psalms, also translated from the "Vulgate," and revised by Cardinal Wiseman; the "Imitation of Christ," "The Spiritual Combat of Father Lorenzo Scupoli," and "The Devout Life of Saint Francis de Sales."

* "The Imitation of Christ," with Introduction by the Venerable F. W. Farrar, D.D. (Methuen and Co. Price 3s. 6d.)

† "Searchings in the Silence." By the Rev. George Matheson, D.D. (Cassell and Co., Limited. Price 3s. 6d.)

‡ "Devotional Library." (Burns and Oates.)

From the same publishers comes a devout work containing "Thirty-three Discourses, with Meditations for the use of the Clergy, Religious, and others,"* full of useful and practical matter, but lacking, like so many of the devotional books of the day, those qualities of fire and true Catholicity which rendered the writings of men like S. Francis de Sales suited to the needs of Christians in all ages and countries.

"God's Birds"† is a prettily-written book for children on the birds mentioned in the Bible, just such a book as children decline to read for themselves, but will love to listen to, hunting up references with happy industry, in one of those difficult hours when the elders decree they are to be kept quiet.

"The History of St. Ignatius Loyola and the Early Jesuits,"‡ compiled by Mr. Stewart Rose, is a valuable work, giving evidence of copious research and vast stores of erudition. A full grasp of the situation and times, Mr. Rose has not, however, attempted, so that, although the book may rank as one of the standard authorities on the life of the saint, it can hardly be accepted as a trustworthy guide in tracing out the intricate series of events which gave rise to the counter-reformation in Europe. The illustrations are rich in historical and archæological interest.

### Recent Fiction.

Mr. Silas Hocking is an adept in that quiet order of novel writing which deals with every-day life, and carries the reader pleasantly through a well-ordered sequence of events to a double wedding, crowned with the rather dubious triumph of a "full churchyard." "A Son of Reuben"§ is a good story of manufacturing life, with the rare merit of containing neither strike nor incendiary. The principal interest centres neither on hero nor heroine, but on the villain of the piece, whose advance from weakness to wickedness is very skilfully portrayed. It were greatly to be wished, however, that he could "dree his weird" in some less conventional manner than in the accident ward of a hospital, with the inevitable result of being nursed by the girl he has jilted.

"The Beechcroft Mystery,"‖ by Carlton Strange, is an ambitious narrative of blood and intrigue, without semblance of unity or pretence at probability. It has all the appearance of having been clumsily patched together out of two distinct sources, so slender is the thread of continuity between the first and second parts. The first or introductory part, dealing with the crimes and subsequent destruction of a gang of French poachers is by far the best part of the book, and is written with some spirit. The speech and actions of English ladies and gentlemen do not lend themselves so readily to the facile pen, and appear, to tell the truth, under strangely distorted guise; this little failing of the sensational writer, very easily condoned if a good plot and strong situations offer a counteracting attraction, renders the "Beechcroft Mystery," in the absence of either, a very dull performance.

Those who want to forget all their surroundings in the excitement of desperate deeds had better look into the "Shafts from an Eastern Quiver,"¶ lately reprinted from the *Strand Magazine*. The illustrations alone, by Mr. Arthur Pearse, are a study in nightmares, as may be seen from the specimen here reproduced, and rouse no small feeling of curiosity to know how these little dilemmas are met in the text, seeing that "nobody seems a penny the worse," what-

* "A Retreat." By the Right Rev. J. C. Hedley. (Burns and Oates.)

‖ "The Beechcroft Mystery." By Carlton Strange. (George Newnes, Limited. 3s. 6d.)

¶ "Shafts from an Eastern Quiver." By Charles J. Mansford. (George Newnes, Limited. Price 3s. 6d.)

ever is done or suffered. The hair-breadth escape business is, perhaps, a trifle overdone, or the colouring is too thick, for all these whirling javelins, cannibals, and precipices fail to move the amount of horror which would be decent under the circumstances. The last story in the book, "The Daughter of Lovetski," is told with more restraint, and conveys a distinct impression of some tragic power and pathos.

A wonderful amount of information is compressed into the weighty annual volume of "Cassell's Family Magazine."* Excellent in type, full of varied entertainment in illustration, no one could turn its leaves without happing instantly on something of interest. The illustrated record of invention, discovery, and science is a specially good feature, and reflects

"Headlong down the abyss." ("Shafts from an Eastern Quiver.")

more marvels in small compass than even the "well-informed person" would care to be examined in. The fiction is decidedly below the level of the more serious articles. A good programme is promised for next year, when a new series at the reduced price of sixpence monthly will be commenced, with stories by J. M. Barrie and L. T. Meade.

* "Cassell's Family Magazine, 1894." (Cassell and Co., Limited).

### Minor Appointment.

General Hospital, Bristol.—Miss Annie Ellis has been made Night Superintendent at this hospital. She was trained at the Royal Portsmouth Hospital, and had charge of a male medical and surgical ward there. Miss Ellis afterwards did private nursing in connection with the Kent Nursing Institute, at Tunbridge Wells; then held the post of Sister of a male surgical ward at the Queen's Hospital, Birmingham, with frequent charge of operating theatre. During the last six months Miss Ellis has had care of a ward at the South-Eastern Hospital, New Cross.

### Christmas in an Irish Hospital.

(By an Irish Correspondent.)

Christmas in hospital! Some people perhaps imagine these words imply everything doleful and depressing, yet this impression, as most readers of The Hospital know, is far removed from the reality.

Before my mind's eye rise pleasant memories of prettily-decorated wards, bright-faced nurses, and happy-looking patients, the latter pale and wan, perhaps, but convalescent, and enjoying life with a zest only known to those who have battled victoriously with serious illness.

I have a very pleasant recollection indeed of a Christmas tea in one of the wards of the Richmond Hospital, Dublin, and of Christmas concerts in those of the City of Dublin Hospital, which seemed to give equal pleasure to nurses, patients, and guests.

Of course there are darker shades in "a hospital Christmas," but so there are also outside hospital walls, the very frost and snow for which young people long meaning bitter suffering to others.

Admitting the existence of these darker shades, it still remains true that it is only when put to the test that people discover how "jolly" they can be under all sorts of trying circumstances. If anyone doubts this let him obtain an invitation to the next Christmas entertainment at the Royal Hospital for Incurables, Donnybrook, and I think there is little doubt that he will come away convinced of its truth.

The regular Christmas festivities in this hospital include dinner in the great dining hall, when the tables, to use a time-honoured phrase, groan beneath the weight of good cheer. Roast beef and plum pudding are, of course, the solid foundations of the feast, embellished by many lighter delicacies.

A full muster of all the patients able to leave their rooms sit down to this Christmas dinner, while those who cannot be present are not forgotten, their share of the good things being conveyed to them by willing hands.

The dining hall is very prettily decorated, as well as all the wards, chiefly by the patients themselves, in honour of Christmas, the wreaths and festoons being allowed to remain undisturbed for ten or twelve days afterwards. There is, indeed, a great deal of friendly rivalry between the different wards, each trying so surpass the others in beauty and novelty of decorations.

A great many governors of the hospital never fail to attend the Christmas dinner in order to assist the regular staff in the onerous, if pleasurable, duty of attendance upon so large a number, and a few specially favoured friends are also recruited as helpers. Such is the Christmas festival proper, which is provided at the expense of the hospital, but during the Christmas holidays other entertainments follow, concerts and teas being arranged by various kind-hearted governors. The concerts are a great pleasure to the patients, and I have never known an audience more easily amused, or who show their enjoyment by more hearty laughter, despite the fact that some are prostrate upon invalid couches, many suffer continually, and none can hope for full restoration to health and strength.

It is good to see the faces light up, the hands beating time to the music, and to hear the storms of applause evoked by some rollicking comic song. These, I think, are generally the most popular portion of the programme, but higher class music and recitations are also thoroughly appreciated, and the kind performers little imagine how much pleasure they bestow, and how much gratitude they awaken, at a trifling cost of time and trouble.

### Where to Go.

Exhibition of Venetian Art.—The New Gallery, Regent Street, Friday, December 21st, and during the season, from 10 to 6.

### Christmas and New Year's Gifts.

The shops have begun to assume a very gay appearance with the approach of the festive season, and fascinating in the extreme are the sights which on all sides greet the eye. The goal attained last year is apparently the starting-point of this to judge by the endless variety of fresh and ingenious novelties that are being displayed. The difficulty of making a decision has become in consequence much greater to the purchaser of to-day than it was a few years ago, when choice was perforce more limited. To the wealthy Christmas shopping is a matter of small moment, a few shillings more or less are of little consequence, but to the majority, whose means are limited, it is a subject of no inconsiderable anxiety. We all like to obtain the best value we can for our money, and to those who would care to see a large assortment of pretty and useful articles at a reasonable cost we should recommend a visit to Messrs. Garrould, of the Edgware Road. Here are all kinds of delightful things varying in price from one shilling upwards. Small Bee clocks in neat red or black cases, and a larger and more costly kind, mounted in gilt and tortoiseshell, of the Louis Quinze period; cases of scissors, so useful to those who sew; books in dainty covers for those who read; and last, but not least, a most attractive display of photograph frames in tapestry, plush, and carved wood.

Nor are the little ones forgotten. Climbing Esquimaux, boxes of soldiers, mechanical toys, are only waiting for a purchaser to bring joy to many a little childish heart. Mr. Reuter (agent for M. Mühlens) has not been idle either, and the most bewitching caskets of Bohemian glass, containing choice perfumes, are now on view at 62, New Bond Street. The newest scent is Maréchal Niel, and is of most exquisite fragrance, recalling the delicate odour of the flower itself. Rhine violets is another delicious speciality of M. Mühlens, which reproduces in a marvellous fashion the subtle and indescribable perfume of the Neapolitan violet. There is a refinement about these perfumes which at once places them in the foremost rank, and we feel sure they only require a trial to secure lasting admiration and patronage. Small boxes, tastefully got up, containing two or four bottles, would make an ideal Christmas present, and one that could not fail to be appreciated.

Those who have leanings in the artistic line should pay a visit to Miss Kate Pragnell, 164, Sloane Street, who is doing some exquisite mineratures on ivory just now. To meet the demand for a similar effect of a less costly nature, this enterprising lady has designed a composite very similar to ivory, at a quarter the cost, and reproduces in colours the most charming impressions. Monochromes in red, brown, and black are in great request. For purity of outline and attention to detail we have seldom seen their equal, and the price is so moderate in comparison with what such things usually cost that they are sure to be in great demand as the season advances. Passing from the ornamental to the useful we invite the reader's attention to Mr. Stacey's, the well-known instrument maker in Newgate Street, where a most fascinating nurse's bag is to be seen. It is quite the latest development of the kind, and is a triumph of skill and ingenuity. The "Mildmay," for so it is called, is made in nut-brown or black cowhide, beautifully finished off with nickel-plated lock and catches, and lined with red leather. Instead of the contents fitting into pockets at the side, they go into a stand, which can be taken out of the bag and placed on the table when required. The convenience of such an arrangement is obvious. This stand is one of the completest things we have seen, as it contains every requisite in the shape of appliances and instruments that can be desired. The fortunate possessor of such an article is to be congratulated, and we can imagine no more delightful present to a district nurse.

### Warm and Useful.

Though London must necessarily always be the centre of attraction where its shops are concerned, yet country firms run it very close in the excellence and variety of their manufactured articles.

Messrs. Fleming, Reid, and Co., the well-known Greenock firm, are in no respect behind their compeers in the production of attractive specialities, and most tempting, both in appearance and usefulness, are the goods they are now offering to the public. Foremost may be noticed their knitted bed-room slippers, which are quite the beau ideal of comfort. They are made in two contrasting shades of Berlin wool mounted on cork soles of the fleeciest and most luxurious softness. One pair in peacock blue and tan-coloured wool is perfectly charming, and is daintily finished off with a bow of peacock blue satin ribbon. These would prove a most acceptable present, and the price is so moderate, that the slenderest purse need fear no strain. Delightful for rheumatic knees are the well-shaped knitted knee-caps. Very fascinating also are the woollen gloves, which can be had in every description of colour and pattern. The knitted underclothing likewise deserves high praise; it is very soft, and does not shrink or become hard in washing. The natural colour of the wool is preserved, and consequently it is entirely free from all deleterious properties in the way of dye. The cholera belts are likely to prove a real boon to all travellers in the East, and are of a most comfortable shape and sufficient thickness to render them useful for any other purpose where warmth and moderate pressure are required. Messrs. Fleming, Reid, and Co. issue a comprehensive catalogue, which likewise includes full instructions for ornamental knitting and crochet, with accompanying illustrations.

Another delightful woollen novelty is a knitted shoulder cape trimmed with chenille, which has been brought out by the Knitted Corset Company (Nottingham). It can be had in all colours, and will be found a real boon to nurses who have draughty corridors to traverse, or who are on night duty, as it can be easily slipped on or off, and is very warm and light.

The Wholesale Supply Company (Market Street, Manchester) have just issued a very complete catalogue of jewellery, silver, and plated goods, and other pretty and useful articles. The watches are especially good value for the money, and are well got up in every way. Christmas cards are more in evidence than ever this season, and Messrs. R. Tuck, Hildesheimer, and Marcus Ward have well sustained their reputation. Fashion changes in this as in other things, but the plain card bearing the monogram and address of the sender, with the usual kindly greeting, will be rather difficult to improve upon.

### A Novel Bandage Cupboard.

All nurses will be fascinated by this most practical and convenient little arrangement for storing bandages for immediate use. The "Shoot" is intended to hang up on the wall, from whence it can be detached in a moment, if necessary, and occupies very little space. Our illustration shows its plan. There are divisions for various sized bandages, a corner for safety pins, and at the side a running roll of strapping. It can be had in two sizes, the larger holding two dozen each of 4-inch, 3-inch, 2½-inch, and 2-inch bandages, and three dozen finger bandages. The price when empty is 17s. 6d., filled with white absorbent bandages, 36s. The smaller size holds one dozen each of the bandages, ranging in sizes from 1 to 3 inches. The price empty is 10s., filled 14s. A more convenient method could hardly be found; the supply cannot run short without due warning, and a bandage of the required size is always ready to hand. Its compactness is a great recommendation, and for casualty rooms, out-patient departments, &c., the "Bandage Shoot," will we feel sure be found very useful. The makers, by whose permission we give the accompanying drawing, are Messrs. Reynolds and Branson, surgical instrument makers, Briggate, Leeds, who are much to be congratulated on having brought out so excellent a little invention.

### The Nurses' Own Diary.

Varied as Messrs. Letts' specialities in diaries were last year, this Christmas they are of a still more comprehensive nature. Most interesting to our readers will be the delightful little "Hospital Nurses' Diary and Handbook." Of a convenient size, strongly and attractively bound in scarlet, its contents will be found no less satisfactory. A complete little history of nursing, useful facts (among which are the fares and distances from London of various health resorts), and postal information precede the diary proper. Notes, memoranda, and cash account conclude a little volume which will prove one of the most inexpensive and welcome of presents among nurses. The "Ladies' Yearbook" contains housekeeping accounts, washing lists, and a variety of other useful matter. The hanging calendars are varied and excellent, and all most moderate in price. Messrs. Letts's address is 3, Royal Exchange, E.C., where nurses can obtain a complete catalogue.

### Everybody's Opinion.

### NURSING IN ASYLUMS.

An Old Asylum Medical Officer writes: There is a nice tone about the letter on this subject in The Hospital for December 8th. It is pleasing to hear asylums praised by one who has had five years' experience in ward work, and who therefore knows much that even medical men find a difficulty in learning. I do not, however, agree with your correspondent when she says that the organization is perfect; but I do believe that general hospitals and kindred institutions are simply "nowhere" when compared to asylums in this respect.

As regards the question referred to in your correspondent's letter, I doubt whether "passes" exist outside the metropolitan area. So, too, with matrons. I was under the impression that asylum matrons, in the old sense, that is, with power to grant leave of absence, &c., were creatures of the past. Perfection in organization, or in anything else, is like that mountain somewhere in Asia, the summit of which has never been reached. An Asylum Nurse may feel sure that perfection has not arrived in asylums as long as passes, matrons, locked doors, airing court walls, and guarded outer gates are in force. If your correspondent will spend her next annual leave in visiting the asylums of Scotland and the North of England, she will probably return with expanded ideas.

### LACKING DISCRETION.

"The Matron of a small Country Hospital" writes: Is not "Matron of a large London Hospital" doing more to bring discredit upon the nursing profession than the nurses whose conduct she "regrets"? Young girls, whether they are nurses or anyone else, are likely to be indiscreet in their remarks, and it would have been a kindly act if the matron had quietly reminded them that their conversation was not suitable for a public conveyance. Whereas their remarks could only have been heard by ten persons at most, the "matron's" repetition of them in The Hospital will be read by hundreds. With all due deference to "The Matron of a large London Hospital," this is how the matter appears to me.

[No doubt the "Matron of a large London hospital" here mentioned knew her London far too well to choose an omnibus as the place in which to publicly reprimand two entire strangers. With regard to the publication of the matter in The Hospital, we think nothing but good will result if it draws the attention of nurses at large to the fact that if they wish their uniform to be respected they must behave with circumspection and discretion when they wear it.—Ed. *T. H.*]

### A CORRECTION.

Miss Peter, Inspector of Nursing, Queen Victoria's Jubilee Institute, writes: Kindly allow me to correct the statement in your issue of December 15th *re* Queen's Nurses at Wolverhampton. There is no branch of the Queen Victoria's Jubilee Institute and no Queen's nurse in that town. The new home opened November 28th, and called "Queen Victoria Institute," has no connection with the Queen's Jubilee Institute, and is for private nurses only. The two district nurses at Wolverhampton live in lodgings in different parts of the town.

### Appointments.

Torbay Hospital, Torquay.—Miss Frances E. G. Ward has been made Matron of this hospital. She was trained at Westminster, where she afterwards held the post of Ward Sister. Miss Ward has been Matron of Dorset County Hospital, and takes many good wishes to her new work.

Accident Hospital, Mansfield, Nottingham.—Miss A. Bradford has been appointed Matron of this hospital. She was trained at Winchester, and held the post of sister there for three years. Miss Bradford was afterwards sister at the Royal South Hants Infirmary, Southampton. We congratulate her on her appointment.

## Figures and Tables

**Figure f1:**
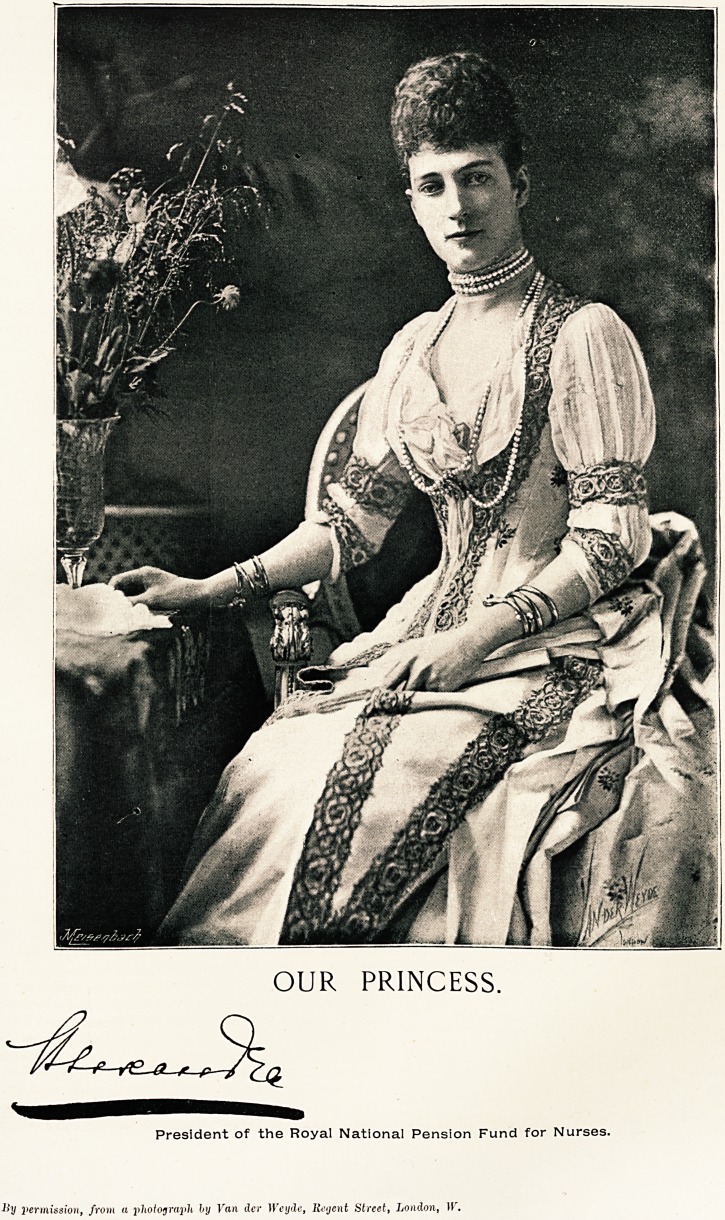


**Figure f2:**
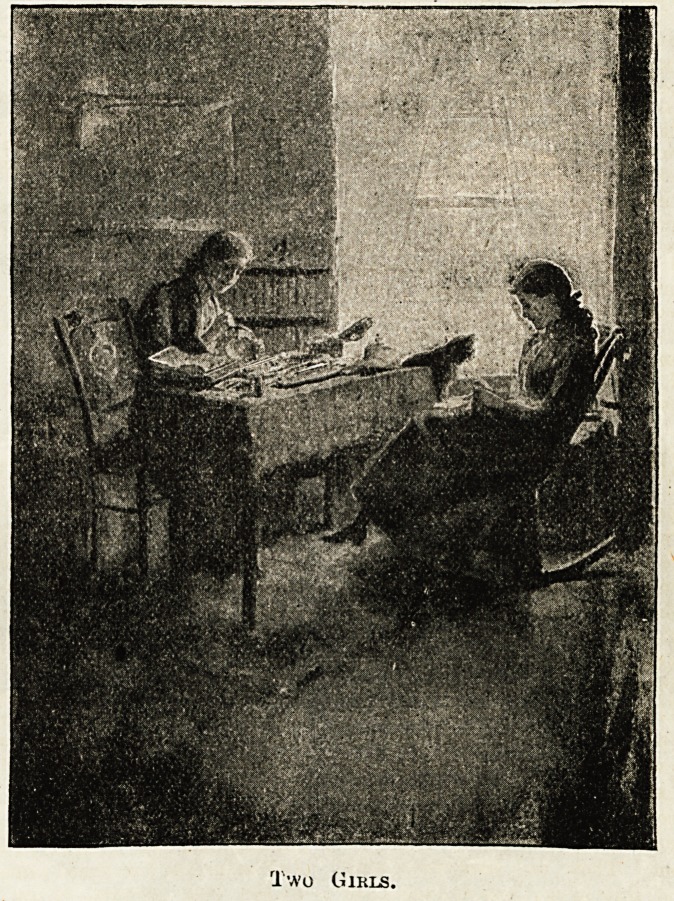


**Figure f3:**
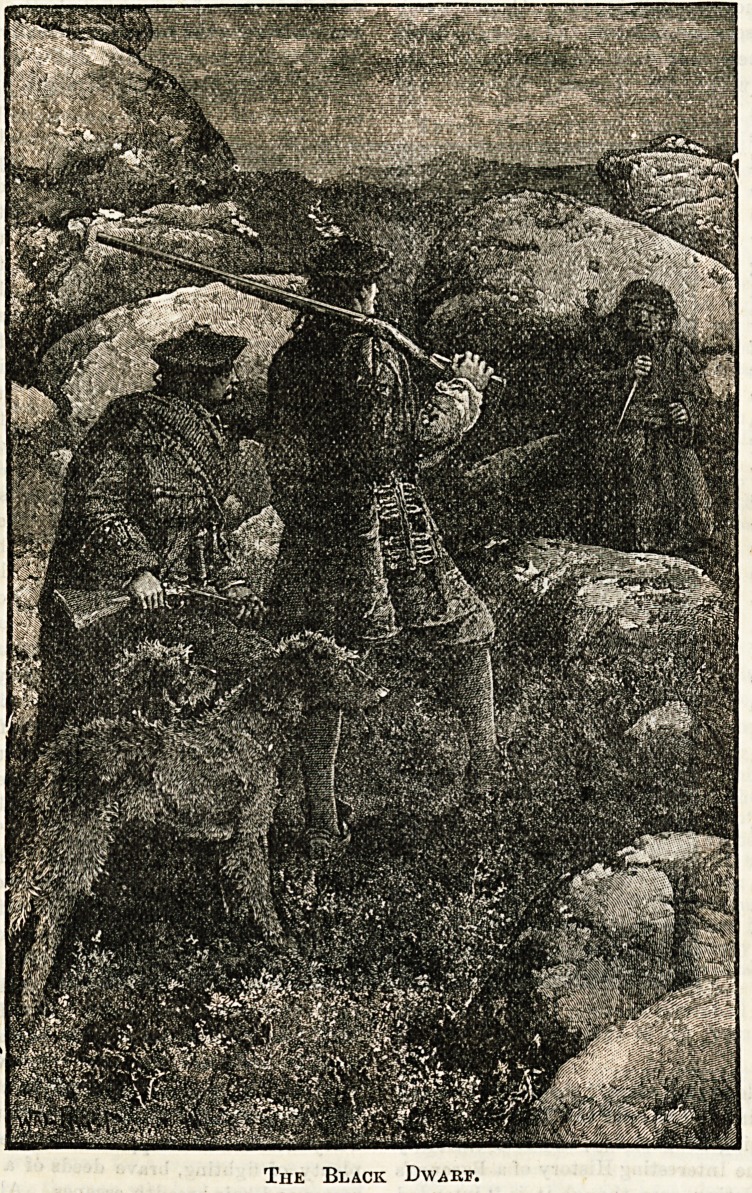


**Figure f4:**
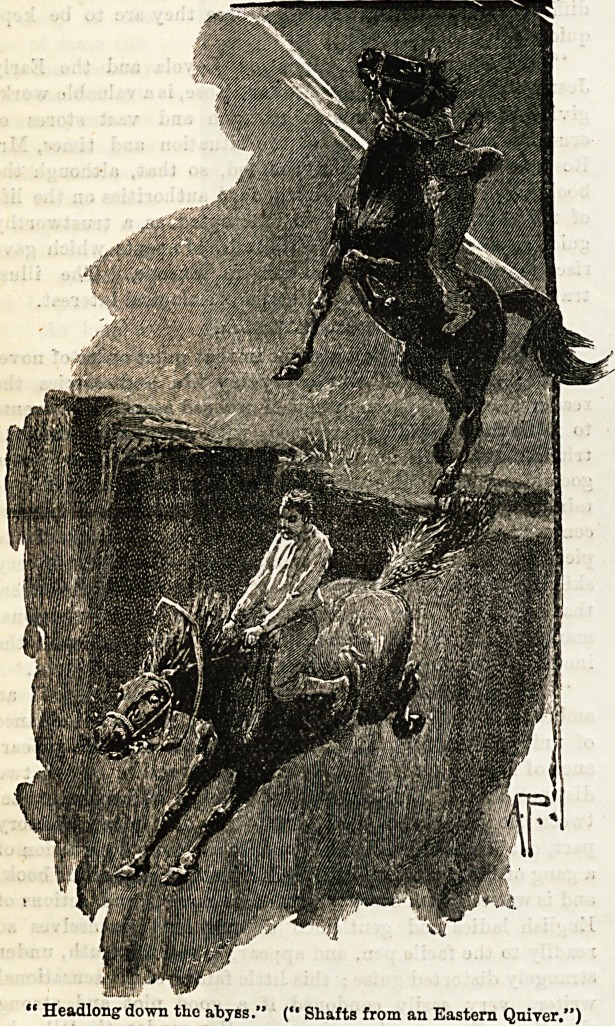


**Figure f5:**
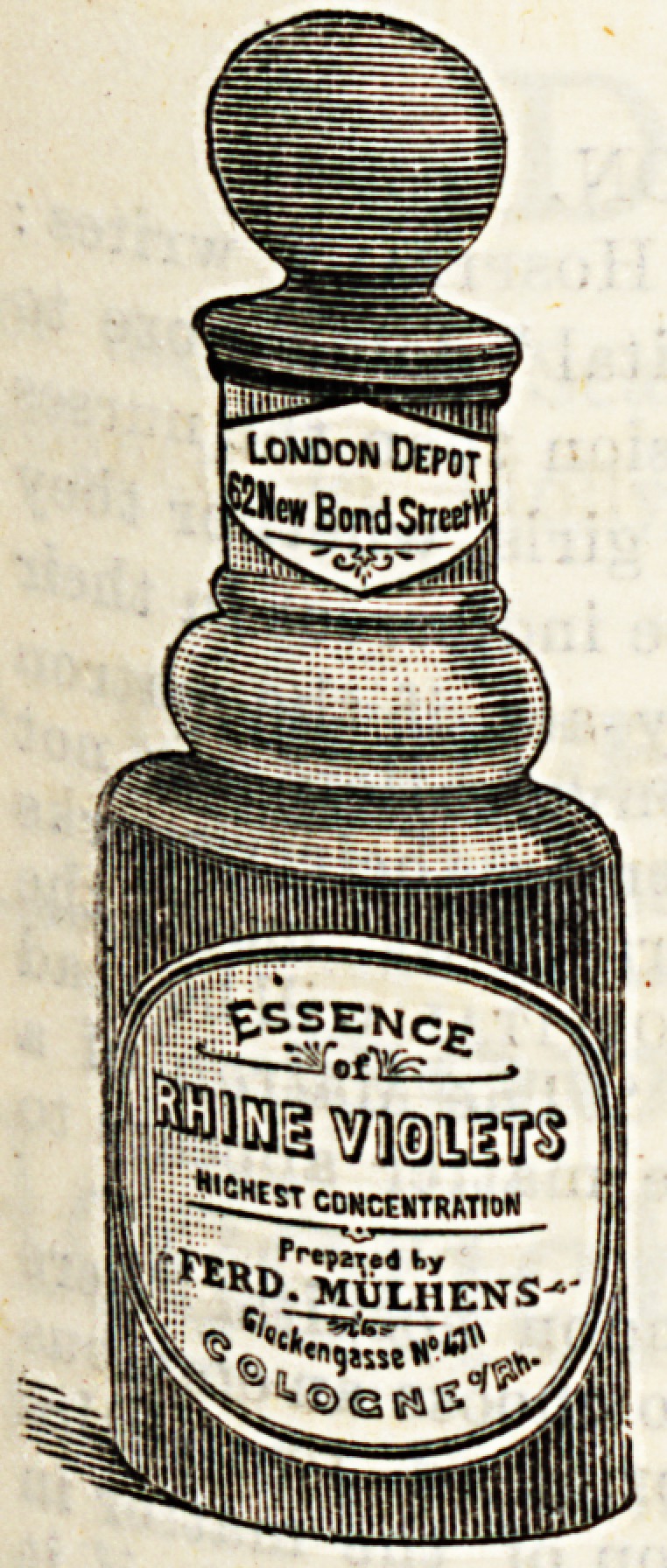


**Figure f6:**
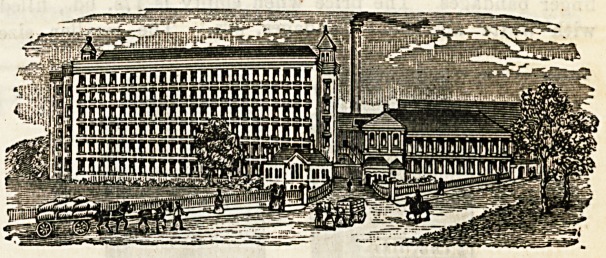


**Figure f7:**
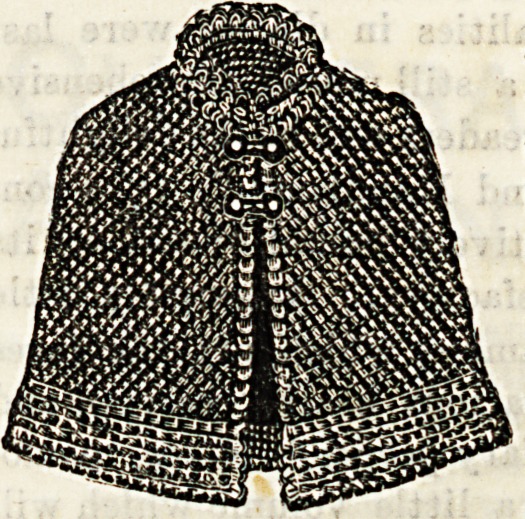


**Figure f8:**
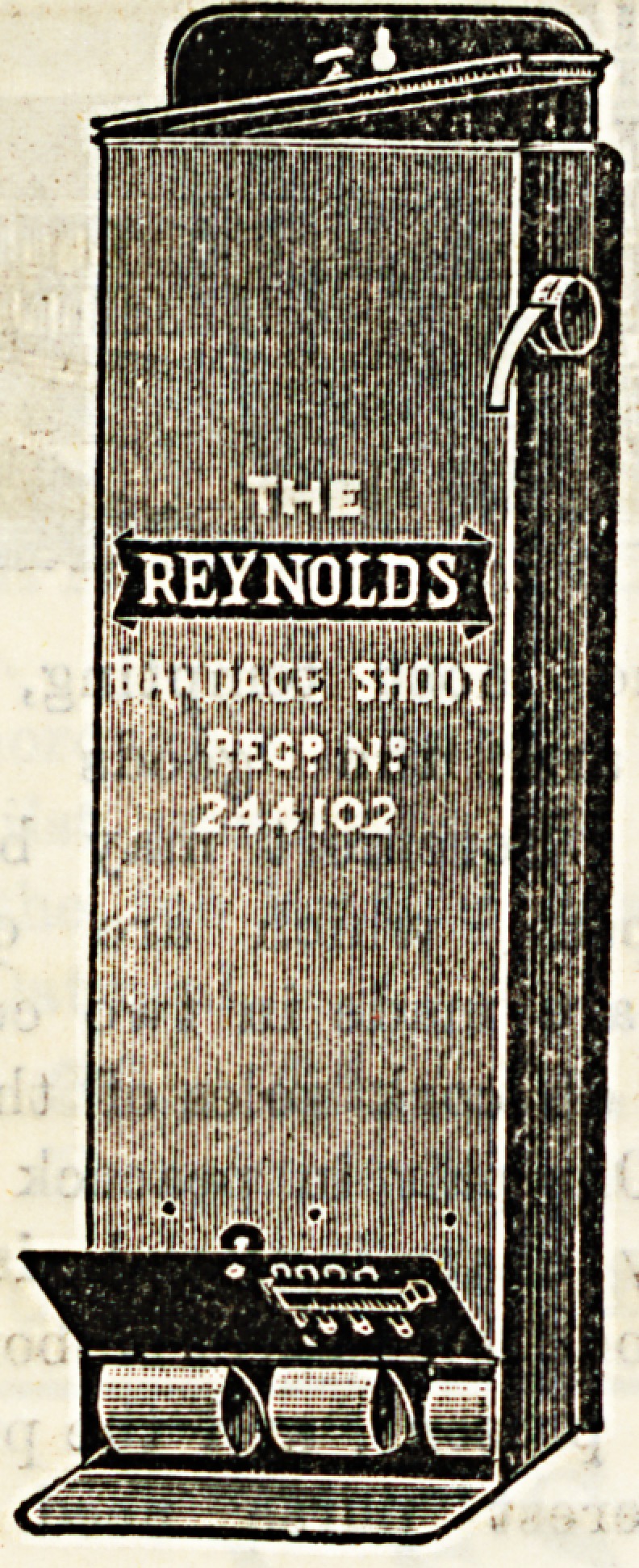


**Figure f9:**